# Concerning the matching of magnetic susceptibility differences for the compensation of background gradients in anisotropic diffusion fibre phantoms

**DOI:** 10.1371/journal.pone.0176192

**Published:** 2017-05-03

**Authors:** Ezequiel Farrher, Johannes Lindemeyer, Farida Grinberg, Ana-Maria Oros-Peusquens, N. Jon Shah

**Affiliations:** 1 Institute of Neuroscience and Medicine – 4, Forschungszentrum Jülich GmbH, Jülich, Germany; 2 Department of Neurology, Faculty of Medicine, RWTH Aachen University, Aachen, Germany; 3 JARA – BRAIN – Translational Medicine, RWTH Aachen University, Aachen, Germany; 4 Institute of Neuroscience and Medicine – 11, Forschungszentrum Jülich GmbH, Jülich, Germany; 5 Department of Electrical and Computer Systems Engineering, and Monash Biomedical Imaging, School of Psychological Sciences, Monash University, Melbourne, Victoria, Australia; Henry Ford Health System, UNITED STATES

## Abstract

Artificial, anisotropic fibre phantoms are nowadays increasingly used in the field of diffusion-weighted MRI. Such phantoms represent useful tools for, among others, the calibration of pulse sequences and validation of diffusion models since they can mimic well-known structural features of brain tissue on the one hand, but exhibit a reduced complexity, on the other. Among all materials, polyethylene fibres have been widely used due to their excellent properties regarding the restriction of water diffusion and surface relaxation properties. Yet the magnetic susceptibility of polyethylene can be distinctly lower than that of distilled water. This difference produces strong microscopic, background field gradients in the vicinity of fibre bundles which are not parallel to the static magnetic field. This, in turn, modulates the MRI signal behaviour. In the present work we investigate an approach to reduce the susceptibility-induced background gradients via reducing the heterogeneity in the internal magnetic susceptibility. An aqueous solution of magnesium chloride hexahydrate (MgCl_2_·6H_2_O) is used for this purpose. Its performance is demonstrated in dedicated anisotropic fibre phantoms with different geometrical configurations.

## Introduction

Diffusion phantoms are highly demanded devices for a broad set of applications in diffusion-weighted (DW) MRI. These applications include the design and calibration of DW MRI experiments [1–5], validation and optimisation of high angular resolution diffusion imaging (HARDI) methods [6–10], tractography algorithms [11–13], validation of diffusion models [14,15] and results from multi-centre studies [16,17]. Artificial, anisotropic diffusion phantoms found in the literature are made of a large variety of materials, e.g., rayon [6,18], hemp [18], polyester [1,5], acrylic fibres [13], glass capillaries [19], plastic capillaries [3,9], hollow fibres produced by co-electrospinning [2,12] and polyamide fibres [4,10]. One of the most frequently used materials is Dyneema^®^ [14,15,18,20,21] (Dyneema^®^ SK75 dtex1760, DSM, Geleen, The Netherlands). These rod-like, hydrophobic, polyethylene fibres have several advantages concerning physical properties compared to other synthetic materials such as nylon and fibre glass, particularly its lower surface relaxivity that results in overall longer relaxation times [20]. This enables diffusion measurements to be performed with higher signal-to-noise ratio (SNR) or longer echo times, which is especially important in the case of strong diffusion weightings and time-dependent studies. Mechanical flexibility is also an advantage when compared to, for instance, rigid capillaries, given that this flexibility facilitates manufacturing of phantoms with a wide range of geometrical configurations. Another advantage of Dyneema^®^ fibres is that they are available with radii below 10 microns, which makes them well-suited for producing phantoms with sufficiently dense diffusion barriers, comparable to that of cellular structures.

Magnetostatic theory predicts that two media of different magnetic susceptibility, *χ*, placed in an external magnetic field introduce distortions to the field distribution in the vicinity of their interface [22]. These distortions, i.e., magnetic field gradients, depend on the difference in magnetic susceptibility, Δ*χ* = *χ*_2_ *- χ*_1_, as well as on the geometric configuration of the media (curvature radii, interface orientation relative to the magnetic field, etc.). Thus, microscopic, susceptibility-induced magnetic field gradients (further on referred to as background gradients) are ubiquitous in NMR and MRI experiments involving magnetically heterogeneous media such as, among others, porous materials [23–25] and biological tissues [26–29]. In particular, background gradients appear as a common feature in anisotropic diffusion phantoms where the magnetic susceptibility of the diffusing liquid is *a priori* different from that of the diffusion-restricting material [4,20,21,30].

As initially demonstrated by Stejskal and Tanner [31], the DW NMR signal in an infinite medium as measured using conventional pulsed gradient spin-echo (PGSE) sequences in the presence of constant background gradients, is influenced by three distinct terms: i) a term dependent on the externally applied field gradient **g**, ii) a term purely dependent on the background gradients **g**_0_, and iii) an interference, cross-term resulting from the interaction between the background and the externally applied gradients. In SE experiments without externally applied field gradients, background gradients act, in the interplay with the diffusion process, as an extra mechanism reducing the measured transverse relaxation time, *T*_2_ [24]. This feature has been successfully used to provide biomarkers of trabecular bone density [27,32,33] or muscle microstructure [34], for example. Recently, computer simulations were used [35] to study the orientation-dependence of *T*_2_ due to background gradients in an array of cylinders under different configurations of Δ*χ*, cylinder packing density and order.

In conventional PGSE, experiments are carried out by measuring the echo attenuation for several strengths of the diffusion-sensitising field gradient **g**, while keeping the sequence timing constant [36]. As a consequence, the signal attenuation due to **g**_0_ becomes merely a multiplicative factor. Special care must be taken though when the strength of **g**_0_ is so large that the signal-to-noise ratio (SNR) becomes critically low for a given echo-time. It has been shown that low values of the SNR in the magnitude signal can lead to a strong underestimation of the apparent diffusion coefficient [37]. On the other hand, the cross-term can lead to more complex implications. It is *a priori* spatially dependent, and therefore it may be cancelled in some regions whereas it may be greatly enhanced in others, even for small **g**_0_ strengths. The effect of the cross-term has previously been measured and empirically modelled by Zhong et al. [38,39] in artificial samples, post mortem and in rat tissue *in vivo*. Their results demonstrate an increase in the echo amplitude for increasing **g**_0_ strengths. This was interpreted as an effect of the regions where **g** was compensated by **g**_0_. The same empirical model was later used by Clark et al. [28] for the analysis of in vivo human brain data, showing similar results. In this context, the same increase in the echo amplitude due to background gradients introduced by the microvessels in the human brain was predicted by Kiselev [40], employing a more realistic model for brain microvasculature.

While background gradients can be exploited to obtain additional structural information on tissue microstructure and orientation [41–45], they can lead to misinterpretation of the results, if they are not properly taken into account in the context of most DW MRI measurements [28,38–40]. Therefore, in many DW MRI experiments, background gradients represent unwanted entities which need to be cancelled. A reduction of the effect of background gradients has been achieved via the design of advanced pulse sequences, as an alternative to the conventional Stejskal-Tanner PGSE sequence [31]. Some of the sequences attempt to reduce the effect of the **g**_0_ term [46,47], whereas others are meant to suppress the effect of the cross-term [46,48]. Nevertheless, a strong underlying assumption in these methods is that the background gradients change slowly throughout space, meaning that each diffusing molecule experiences the same constant background gradient during the observation time. Ref. [46] provides a comprehensive review on this topic.

A different approach for the suppression of background gradients is susceptibility matching. Its aim is to reduce, or ideally entirely eliminate, the difference in magnetic susceptibilities within the sample. This approach has been previously used in NMR spectroscopy studies [49] to reduce the line broadening in NMR spectra. More recently susceptibility matching was carried out in a polyamide fibre phantom. In their work, Laun et al. [4] used a solution of 83 grams of sodium chloride (NaCl) per kilogram of distilled water, in order to match the susceptibility of the solution to that of the polyamide fibres, which is 0.37 ppm more diamagnetic than distilled water.

The aim of this work is to design a strategy to suppress unwanted background gradients by matching the susceptibility of the liquid to that of the fibre material. In order to achieve this, the effects of background gradients on diffusion metrics in anisotropic phantoms constructed with Dyneema^®^ fibres and different fibre configurations are assessed. We investigate the influence of background gradients on diffusion tensor imaging (DTI) invariant metrics, as well as on two methods for the analysis of HARDI data, namely constrained spherical deconvolution (CSD) [50] and q-ball imaging (QBI) [51]. Three types of dedicated phantoms were used for this purpose: i) bulk phantoms, i.e., test vials containing the solution of interest embedded in a cylindrical phantom; ii) parallel-fibre phantoms, i.e., showing single-modal diffusion profiles; iii) a phantom with two fibre populations crossing at the right angle, i.e., showing multi-modal diffusion profiles.

## Theory

The PGSE signal attenuation for an infinite, homogeneous and anisotropic medium with uniform background gradients **g**_0_ = *g*_0_**n**_0_, in the presence of a diffusion sensitising, pulsed field gradient **g** = *g***n**, is given by [24,36]

$$S\left(b,n\right)=S_{0}\text{exp}\left[-\left(\underset{\text{external}}{\underset{︸}{b\cdot n^{\text{T}}Dn}}+\underset{\text{cross term}}{\underset{︸}{b_{\text{c}}\cdot n^{\text{T}}Dn_{0}}}\right)\right],$$ (1)

where *S*_0_ is the DW signal in the absence of diffusion weighting, **D** is the symmetric, positive-definite diffusion tensor, and **n** and **n**_0_ are the unit vectors along the diffusion sensitising and background gradients, respectively. The *b*-value due to the applied gradient is given by $b=\gamma^{2}\int_{0}^{T_{\text{E}}}{\left(\int_{0}^{t}g\left(t\prime \right)dt\prime \right)}^{2}dt$, whereas the *b*-value related to the cross-term can be shown to be $b_{\text{c}}=2\gamma^{2}\int_{0}^{T_{\text{E}}}\left(\int_{0}^{t}g\left(t\prime \right)dt\prime \right)\left(\int_{0}^{t}g_{0}\left(t\prime \right)dt\prime \right)dt$, with *g*(*t*) and *g*_0_(*t*) denoting the gradients waveforms, *T*_E_ the echo time and *γ* the gyromagnetic ratio. For the conventional Stejskal-Tanner PGSE sequence *b* = *γ*^2^*g*^2^*δ*^2^(Δ − *δ/3*) and *b*_*c*_ = *γ*^2^*gg*_0_*δ*(Δ − *δ/3*)[*T*_*E*_ − (Δ − *δ*)/2], where *δ* and Δ are the time duration and separation of the field gradient pulses, respectively [40]. Eq (1) corresponds to the conventional case in which the DW signal is acquired for several strengths of the field gradient while keeping the sequence timing constant. Thus, *S*_0_ includes a diffusion weighting term that is purely dependent on the background gradients, which effectively acts as an extra mechanism inducing transverse relaxation.

In the framework of DTI analysis, estimation of tensor elements is performed by regressing

$$S\left(b,n\right)=S_{0}\text{exp}\left[-\left(bn^{\text{T}}D_{\text{app}}n\right)\right],$$ (2)

to the measured DW signal. In Eq (2) the apparent diffusion tensor, **D**_app_, is introduced to emphasize the possible bias in **D** arising as a consequence of neglecting the cross term and the dependence on the pulse sequence and timing parameters employed. It is worth noticing that the diffusion weighting term related to background gradients only will not significantly bias the estimation, provided that the SNR is high enough. In case *S*_0_ is close to the noise floor, special care must be taken in order to avoid bias in the tensor elements. Conventionally, regression of Eq (2) to the experimental data is performed via a least-squares minimisation approach. However, for low SNR experiments the maximum likelihood estimator is preferred in order to ensure unbiased estimation [52].

### System with cylindrical geometry

For a system of perfectly aligned cylinders, the background gradients only have components in the radial direction, i.e., perpendicular to the axis of symmetry [40]. Therefore, the DW signals acquired utilising gradient directions parallel (**n**_||_) and perpendicular (**n**_⊥_) to the axis of symmetry can be calculated using Eq (1), yielding

$$\begin{array}{l} \begin{array}{cccc} S\left(b,n_{\left|\right|}\right) & = & S_{0}\text{exp}\left[-bD_{\left|\right|}\right] & \text{and} \end{array}\\ \begin{array}{ccc} S\left(b,n_{⊥}\right) & = & S_{0}\text{exp}\left[-\left(b+b_{\text{c}}n_{⊥}^{\text{T}}n_{0}\right)D_{⊥}\right] \end{array}, \end{array}$$ (3)

where *D*_||_ and *D*_⊥_ denote the diffusivities in the direction parallel and perpendicular to the axis of symmetry, respectively. While the estimation of *D*_||_ using Eq (2) would remain unbiased, the dot product $n_{⊥}^{\text{T}}n_{0}$ can take positive (parallel), negative (anti-parallel) or zero values. In such systems, this term may also vary throughout space. More importantly, for densely packed systems the gradient may be non-linear in the length-scale of the molecular root mean square displacement. Hence, the estimation of *D*_⊥_ via Eq (2) can potentially be biased.

## Material and methods

### Selection of the appropriate compound

Pure water is diamagnetic with a magnetic susceptibility of about *χ*_water_ = -9.05ppm [53]. The susceptibility values of typical compounds range from strong paramagnetism (positive values) to comparably small diamagnetism (negative values). In particular, materials soluble in water with a magnetic susceptibility lower than that of water are rare. The susceptibility of the fibre material Dyneema^®^ is approximately -10ppm [20], which is about 1ppm below water. Promising candidates for lowering the susceptibility value of aqueous solutions are salts since they are typically good solutes in water and many of them are strongly diamagnetic. Unfortunately, numerous salts also exhibit high electrical conductivity [54]. Hence compounds such as NaCl or KCl are poor candidates for approaching high susceptibility differences due to their high electrical conductivity and their consequently high radiofrequency absorption. Magnesium Chloride (MgCl_2_) is diamagnetic with a susceptibility of *χ* = 4π · (*ρ/M*) · *χ*_m_(cgs) = −14.6ppm, *χ*_m_(cgs) = -47.4cm^3^/mol [55], where *ρ* is the density and *M* represents the molar mass. It is also highly soluble in water with up to 552 g/l at 20°C [55] and its conductivity at a mass percentage of 25% is roughly half that of e.g. NaCl [54]. The hydrated form, magnesium chloride hexahydrate (MgCl_2_·6H_2_O) can be easily mixed with water. Accurate values of the magnetic susceptibility of different MgCl_2_·6H_2_O concentrations in distilled water are to our knowledge not reported in literature.

### Bulk experiments

All MRI experiments were carried out in a whole-body 3T Siemens MAGNETOM Trio scanner (Siemens Medical Systems, Erlangen, Germany). The body coil was used for radiofrequency transmission and a 12-channel phased-array head coil was used for signal reception. All experiments were performed at room temperature. The gradient system provided a maximal gradient strength of 40 mT/m.

**Fig 1:**
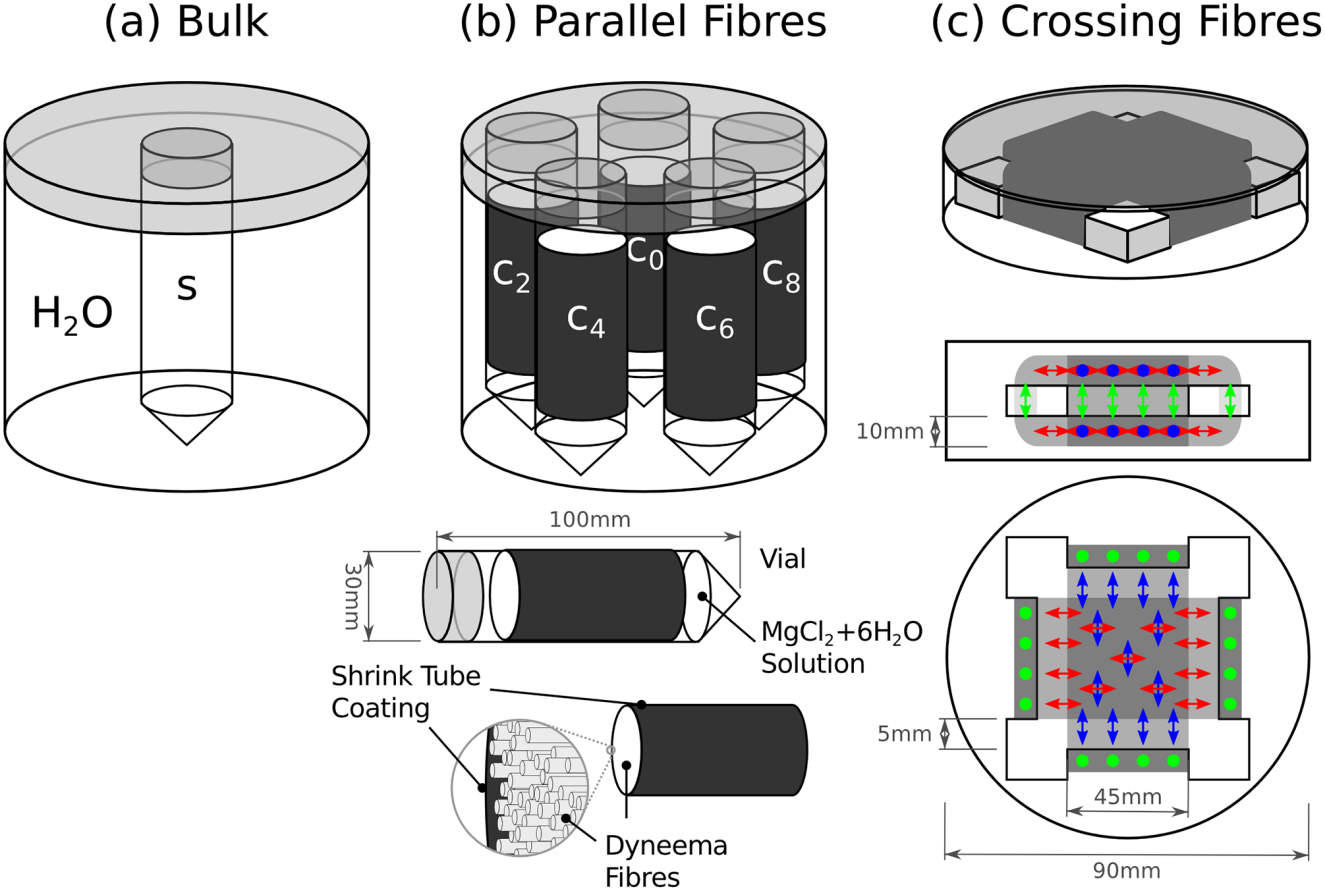
Schematic representation of the three phantom types used in this study. (a) Bulk phantoms, consisting of a plastic vial containing the concentration of interest, embedded in a cylindrical PET phantom filled with distilled water. Field distortions around the vial where used to assess the magnetic susceptibility. (b) Parallel-fibres phantoms containing concentrations *c*_0_, *c*_2_, *c*_4_, *c*_6_ and *c*_8_ in the interstitial space. Shrinking tubes were used to compress the fibres and keep them parallel. (c) Crossing-fibre phantom, with fibres wound around a Perspex platform and crossing at right angle.

A set of 9 solutions was prepared with increasing MgCl_2_·6H_2_O concentrations, *c*_*v*_ (*v* = 0,…,8), in distilled water in order to assess bulk NMR properties (Table 1). Test vials were filled with these solutions and embedded, one at a time, into a cylindrical polyethylene terephthalate (PET) plastic phantom (10 cm diameter, 14 cm height) filled with distilled water (Fig 1a).

**Table 1:** Bulk concentrations of MgCl_2_·6H_2_O.

tube number (*v*)	0	1	2	3	4	5	6	7	8
concentration (*c*) [mol/l]	0	0.74	1.35	1.87	2.30	2.68	3.01	3.30	3.55

Concentrations of MgCl_2_·6H_2_O in distilled water used in the assessment of the bulk and the parallel-fibre phantoms properties.

**Fig 2:**
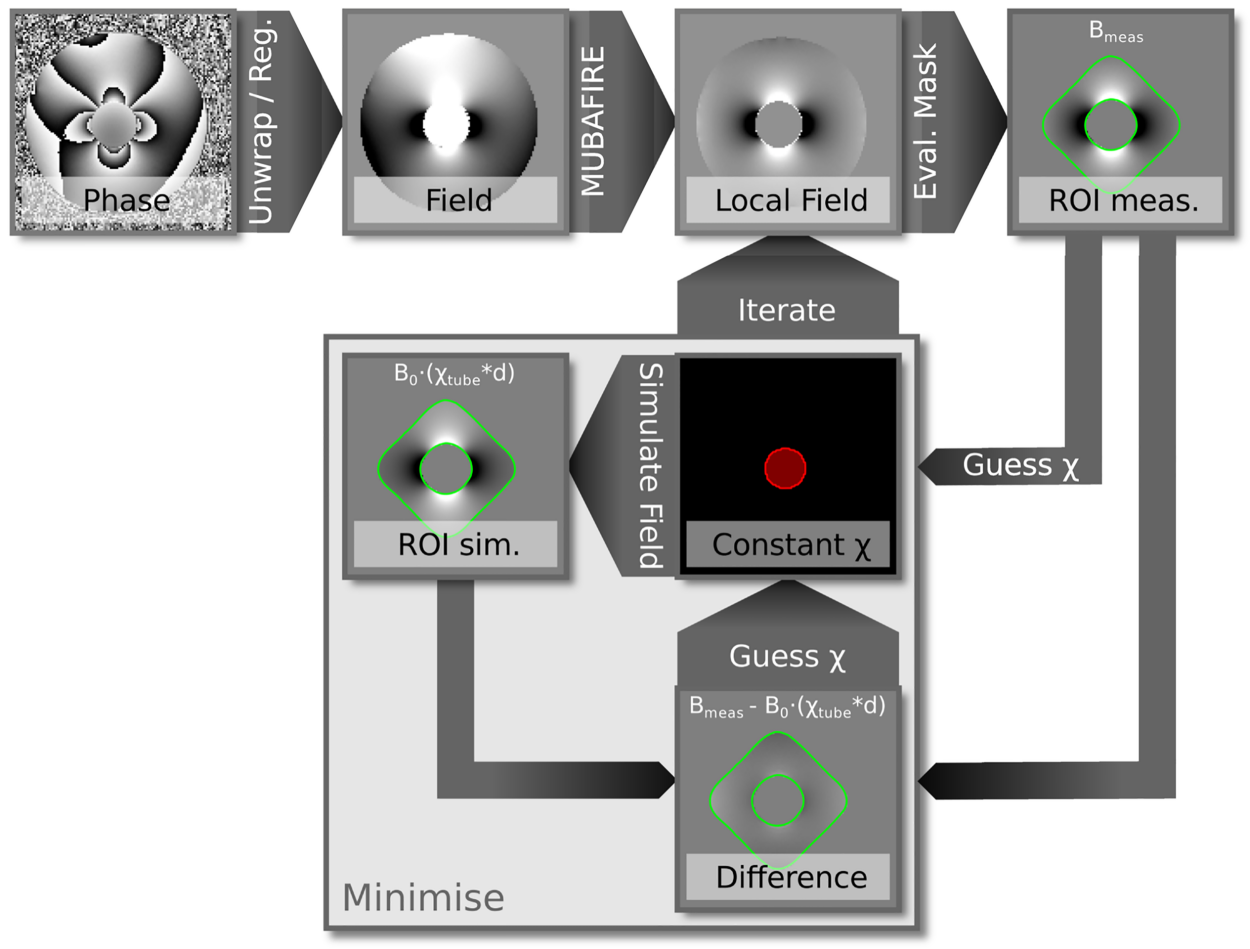
Data processing workflow for the assessment of the bulk magnetic susceptibility values. Starting from the top left, the raw phase is spatially unwrapped, the echoes are realigned and the field map is estimated by linear regression; background fields are removed using MUBAFIRE and a reduced ROI for evaluation is selected; setting a constant susceptibility value inside of the vial volume, the difference between the hereby generated and the measured field distortion is minimised; the process is iterated three times in order to take the estimated vial field into account in the background correction.

Magnetic susceptibility is assessed via the field distortions generated by the cylindrical tube in the surrounding water as in quantitative susceptibility imaging (QSM, [56,57]). The strongest distortions can be achieved with the cylinder orientation perpendicular to the static magnetic field [58]. The field distribution inside the phantom is estimated by linear regression on the phase data acquired with a multiple-echo, gradient-echo (GRE) sequence at 1mm isotropic resolution; matrix-size, 128×128×160; flip-angle, 14°; TE = 3ms (8 echoes, ΔTE = 4ms; bandwidth, BW = 501 Hz/pixel); repetition time, TR = 60ms; using monopolar readout. Automated threshold- and morphology-based segmentation is applied to the signal magnitude in order to generate masks for the vial content, *m*_v_, and the water volume, *m*_w_. An evaluation area surrounding the vial, *m*_e_, is defined by expanding the vial mask. Our in-house software, MUBAFIRE [59], is used to correct for field distortions originating from sources outside of the phantom. The susceptibility distribution *χ*_*v*_ inside the vial is assumed to be constant as the liquid is homogeneous. Furthermore, setting *χ* to 0 within the water mask defines *χ*_water_ as the reference offset. The difference between the measured field and the one generated by dipole convolution [60] of *χ*_*v*_ within a cylindrical water mask, *m*_e_, around the test vial is evaluated (Fig 2) and minimised for *χ*_*v*_:

$$\underset{\chi_{\text{v}}}{\text{min}}{\left\|m_{\text{e}}\cdot \left(B_{\text{meas}}-B_{0}\cdot \left[\chi_{\text{v}}m_{\text{v}}⊗d\right]\right)\right\|}_{2},$$ (4)

where *d* is the appropriate dipole kernel [61]. The internal stray field of the vial can bias the background field correction. In order to compensate for this effect, background correction and susceptibility estimation are iterated three times. In each iteration, the estimated vial field of the previous iteration is subtracted during background correction. The whole procedure is illustrated in Fig 2. The accuracy of the susceptibility estimate is indicated by the relative difference between background-corrected field and the field generated by the determined susceptibility *χ*_v_, evaluated within *m*_e_. Error values are defined as the mean-corrected field difference divided by the mean-corrected measured field.

For the measurement of the bulk *T*_1_ relaxation times all vials were placed in the so-called “revolver phantom”. Measurements were performed with the TAPIR sequence [62]. Protocol parameters were: TR = 25 ms; flip-angle, 20°, inversion-times, 20 ≤ TI ≤ 4180 ms; BW = 320 Hz/pixel; voxel size, 1×1×4 mm^3^; matrix-size, 200×200×1. Data were processed using in-house Matlab scripts (Matlab 2015a, The MathWorks, Natick, MA, USA).

The bulk *T*_2_ and proton density (PD) for all vials are estimated with the help of a 2D spin-echo multi-contrast sequence provided by the manufacturer, using the following protocol parameters: 32 contrasts with an inter-echo time spacing of 50 ms; TR = 10^4^ ms; number of averages, AVGs = 3; BW = 781 Hz/pixel; voxel size, 2×2×10 mm^3^ and matrix-size, 96×128×1. The echo attenuation, *S*(*T*_E_), is assumed to be monoexponential

$$S\left(T_{\text{E}}\right)=S_{0}\text{exp}\left(-\frac{T_{\text{E}}}{T_{\text{2}}}\right),$$ (5)

where *S*_0_ is the signal at *T*_E_ = 0. *S*_0_ and *T*_2_ are estimated voxel-wise via non-linear least-squares using the Nelder-Mead algorithm with in-house Matlab scripts. Finally the PD for each concentration *c*_*v*_ is evaluated as PD_*v*_ = *S*_0,*v*_/*S*_0,0_, with *S*_0,*v*_ being the corresponding signal at TE = 0 [14,21].

Measurement of the bulk diffusion coefficient, *D*, for all concentrations *c*_*v*_, was carried out using a 2D twice-refocused SE (TRSE) EPI sequence with bipolar diffusion weighting gradients provided by the manufacturer [63]. Protocol parameters were: TR/TE = 8000/112 ms; AVGs = 16; BW = 1628 Hz/pixel; voxel-size, 2×2×10 mm^3^; matrix-size, 96×128×1; 16 *b*-values, [0:0.2:3.0] ms/μm^2^ along a single gradient direction; GRAPPA acceleration factor 2, with 24 reference lines. All DW MRI experiments in this work are performed such that different *b*-values are achieved by varying the gradient strength *g* while keeping the timing parameters *δ* and Δ constant. Considering the isotropic diffusion of this case, *S*_0_ and *D* are estimated voxel-wise via non-linear maximisation of the log-likelihood function as described in the section *Maximum likelihood estimation of diffusion parameters*.

### Parallel-fibre phantom

All fibre phantoms were constructed using Dyneema^®^ fibres (rod-like fibres with a radius of approximately 8 μm) [14,20,21]. Five parallel-fibre phantoms were built using bundles of Dyneema^®^ fibres aligned in parallel. Each bundle was placed inside a shrinking tube approximately 5 centimetres wide [14,20] and afterwards heated to shrink it to a final diameter of about 2.5 centimetres. The fibre density for each phantom was 0.64 ± 0.02. Each phantom was then embedded in plastic vials that were filled with MgCl_2_·6H_2_O concentrations *c*_0_, *c*_2_, *c*_4_, *c*_6_ and *c*_8_ (Table 1), in such a way that the interstitial space between the fibres was filled via capillary force. Finally, all vials were assembled in a cylindrical PET plastic container (10 cm diameter, 14 cm height) filled with distilled water for the subsequent MRI experiments (Fig 1b).

Experiments were carried out for five different orientations of the fibre bundles with respect to the static field **B**_0_ at *θ* = 0°, 22.5°, 45°, 67.5° and 90°. The TRSE sequence was used. Protocol parameters were: TR/TE = 4200/82 ms; AVGs = 10; BW = 1563 Hz/pixel; voxel-size, 2×2×2 mm^3^; matrix-size, 80×128×36; 5 *b*-values, [0:0.25:1.0] ms/μm^2^ along 30 gradient directions; GRAPPA acceleration factor 2, with 24 reference lines; phase-encoding direction right-to-left. An extra non-DW volume with opposite phase-encoding direction was acquired for the correction of EPI distortions.

Eddy-current and EPI distortion correction is applied to all volumes with the help of the EDDY tool available in FSL [64–66]. Subsequently, the diffusion weighting directions are reoriented according to the transformation matrices obtained in the former step [67]. Spatial smoothing with a Gaussian convolution kernel (full-width-half-maximum 2.3 mm) is applied to the images. Given the anisotropic nature of diffusion in this case, *S*_0_ and the apparent diffusion tensor **D**_app_ are estimated as described in the section *Maximum likelihood estimation of diffusion parameters*.

### Crossing-fibre phantom

The crossing-fibre phantom was built by tightly winding the fibres around a Perspex support (Plexiglas^®^) in order to keep the geometrical shape (Fig 1c). Several layers of fibres were stacked in perpendicularly alternating directions in such a way that the resulting thickness was approximately 10 mm [21]. The fibre density for this phantom was 0.68 ± 0.02. The whole setup was immersed in a PET plastic cylindrical container (10 cm diameter, 4 cm height). In order to assess the effect of internal gradients, two different cases were considered:

1. *Unmatched* case. The phantom was first filled with distilled water and the whole set of MRI experiments was carried out.
2. *Matched* case. The phantom was drained and refilled with the matching MgCl_2_·6H_2_O concentration (see section Results: *c*_3_ = 1.87 mol/l with *χ*_3_ ≈ -1.01 ppm), repeating the same MRI experiments.

In both cases the phantom was placed in a vacuum chamber for four hours in order to remove remaining air bubbles.

Experiments were carried out for three orientations of the phantom such that the angle, *θ*, between one of the fibre populations and **B**_0_ was *θ* = 0°, 22.5° and 45°. Thus, the angle between the remaining fibre population and **B**_0_ was 90°–*θ* = 90°, 67.5° and 45°, respectively. Protocol parameters were: TR/TE = 5000/113 ms; AVGs = 8; BW = 1563 Hz/pixel; voxel size, 2×2×2 mm^3^; matrix size, 88×128×14; GRAPPA acceleration factor 2, with 24 reference lines; phase-encoding direction right-to-left. An extra non-DW volume with the opposite phase-encoding direction was acquired for the correction of EPI distortions. The applied *b*-values were: *b* = 0, 1.0 ms/μm^2^ (*unmatched*) and *b* = 0, 2.0 ms/μm^2^ (*matched*) along 64 gradient directions. The different *b*-values for the *unmatched* and the *matched* cases were chosen so as to maintain the comparability of the products (*bD*)_*unmatched*_ and (*bD*)_*matched*_. The non-DW signal, *S*_0_, and the apparent diffusion tensor **D**_app_ are estimated voxel-wise as described in the section *Maximum likelihood estimation of diffusion parameters*.

An evaluation of the performance of the CSD and QBI methods in assessing fibre directionality is also carried out. The normalised fibre orientation distribution (FOD) defined in the framework of CSD and the orientation density function (ODF) in the case of QBI are obtained voxel-wise using the toolbox ExploreDTI [68]. Results are generated using maximum spherical-harmonic order *l*_max_ = 6. Fibre orientations are assessed via the local maxima of the FOD and ODF, using a Newton optimisation algorithm [69]. A threshold equal to 10% of the maximum peak is used to reject spurious peaks. The deviation angle, Δ*α*, between the predicted and the underlying, mean fibre direction is evaluated as

Δαp=1N∑i=1N|cos−1(M⋅pmp,i|Mp|)|, (6)

where *p* = 1, 2 denotes the peak number, *N* is the number of voxels in the ROI, **m**_*p*,*i*_ is the voxel-wise unit vector pointing in the *p*^*th*^ fibre direction and **M**_*p*_ is a vector pointing along the average *p*^th^ fibre direction, M=p∑j=1Nmp,j.

### Maximum likelihood estimation of diffusion parameters

The maximum likelihood estimator (ML) is used in order to avoid bias in the diffusion parameters due to the low SNR observed in some of the DW experiments described above [52]. The ML estimator is written as:

$$\text{arg }\underset{\beta }{\text{max}}\left[\text{log}p_{X}\left(X|S\left(\beta \right),\sigma \right)\right],$$ (7)

where *S*(**β**) is the signal model (Eq (2)), **β** is a vector containing all parameters of interest, **X** is a vector containing the measured signals *x*_*i*_, *p*_x_ = **∏**_*i*_*p*(*x*_*i*_|*S*(**β**), σ) denotes the joint probability density function for *x*_*i*_, and *p*(*x*_*i*_|*S*(**β**), σ) is the Rice distribution with background noise parameter σ [52]. Here, the parameter σ is estimated following the method proposed by Aja-Fernández et al. [70]. For each case, maximisation of Eq (7) is performed using the Nelder-Mead algorithm with in-house Matlab scripts.

Further evaluation of the DTI invariant scalar maps, namely axial (AD), representing *D*_||_, radial (RD), representing *D*_⊥_, and mean (MD) apparent diffusivities as well as fractional anisotropy (FA), is performed as described elsewhere [36]. Furthermore, a ROI analysis is carried out for all metrics. The brackets 〈*A*〉 denote the average of a given parameter *A* over a ROI, whereas the standard deviation is calculated as $\sigma_{A}=\sqrt{\sum_{i=1}^{N}{\left(A_{i}-\langle A\rangle \right)}^{2}/\left(N-1\right)}$, where *N* is the number of voxels in the ROI.

## Results

### Bulk experiments

**Fig 3:**
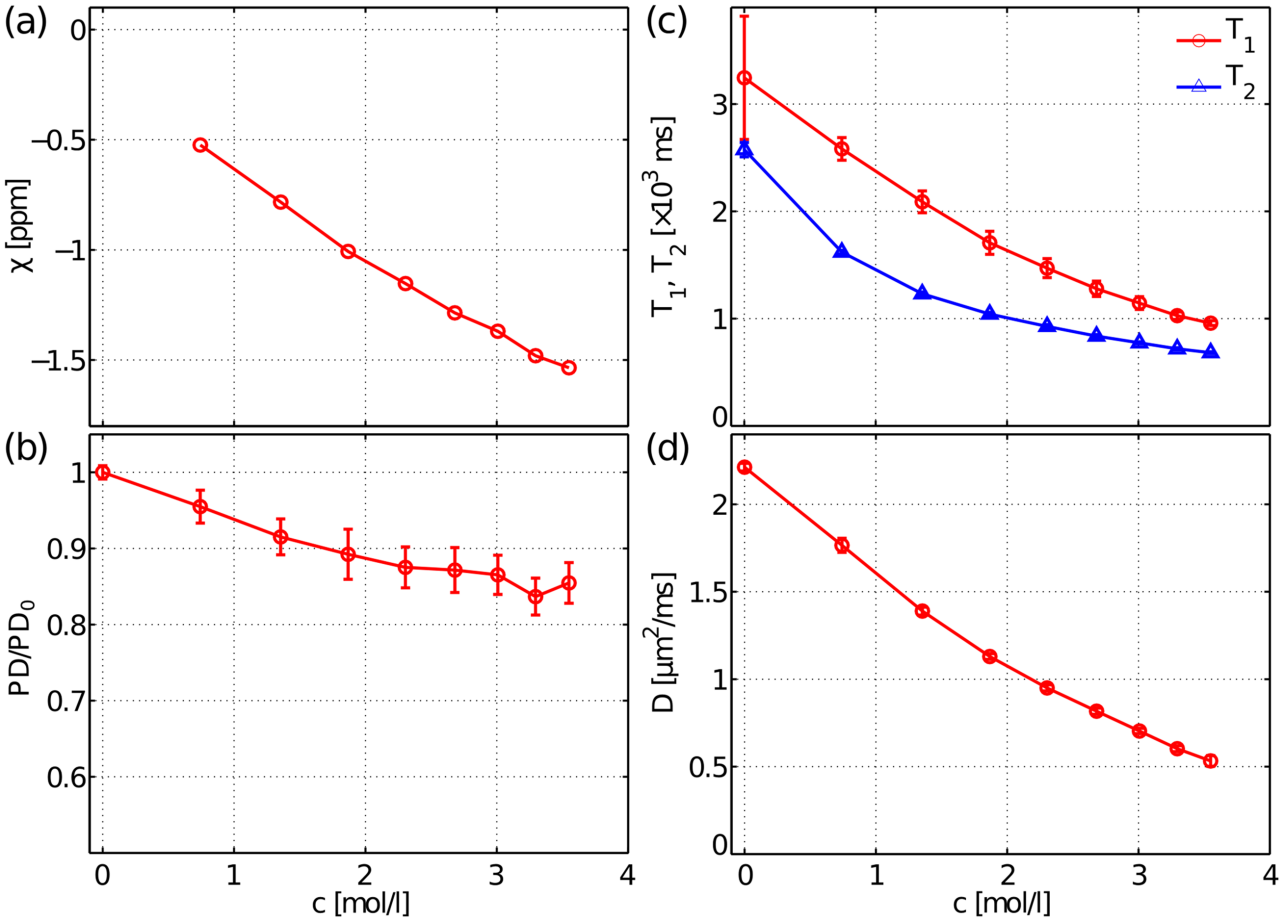
Concentration dependence of bulk NMR quantities. Magnetic susceptibility (a), proton density (b), relaxation times *T*_1_ and *T*_2_ (c) and diffusion coefficient (d).

Fig 3 illustrates the bulk magnetic susceptibility difference with respect to distilled water, *χ* (a), the relative proton density PD (b), the relaxation times *T*_1_ and *T*_2_ (c), and the bulk diffusion coefficient *D* (d), for the 9 concentrations of MgCl_2_·6H_2_O (Table 1). The susceptibility estimation shows high precision with a relative field error of around 2%. Increasing the concentration of MgCl_2_·6H_2_O leads to a monotonous decrease of all parameters. In particular, for the concentration *c*_3_ (1.87 mol/l) the magnetic susceptibility is *χ*_3_ ≈ -1.01 ppm, which practically matches the susceptibility of the Dyneema fibres (Fig 3a) [20]. Concomitant with that change there is a reduction in the proton density, in both relaxation times and in the diffusion coefficient. For the concentration *c*_3_ the following values are observed: PD = (0.83±0.03), *T*_1_ = (1700±100) ms, *T*_2_ = (1040±20) ms and *D* = (1.13±0.02) μm^2^/ms.

### Parallel-fibre phantom

**Fig 4:**
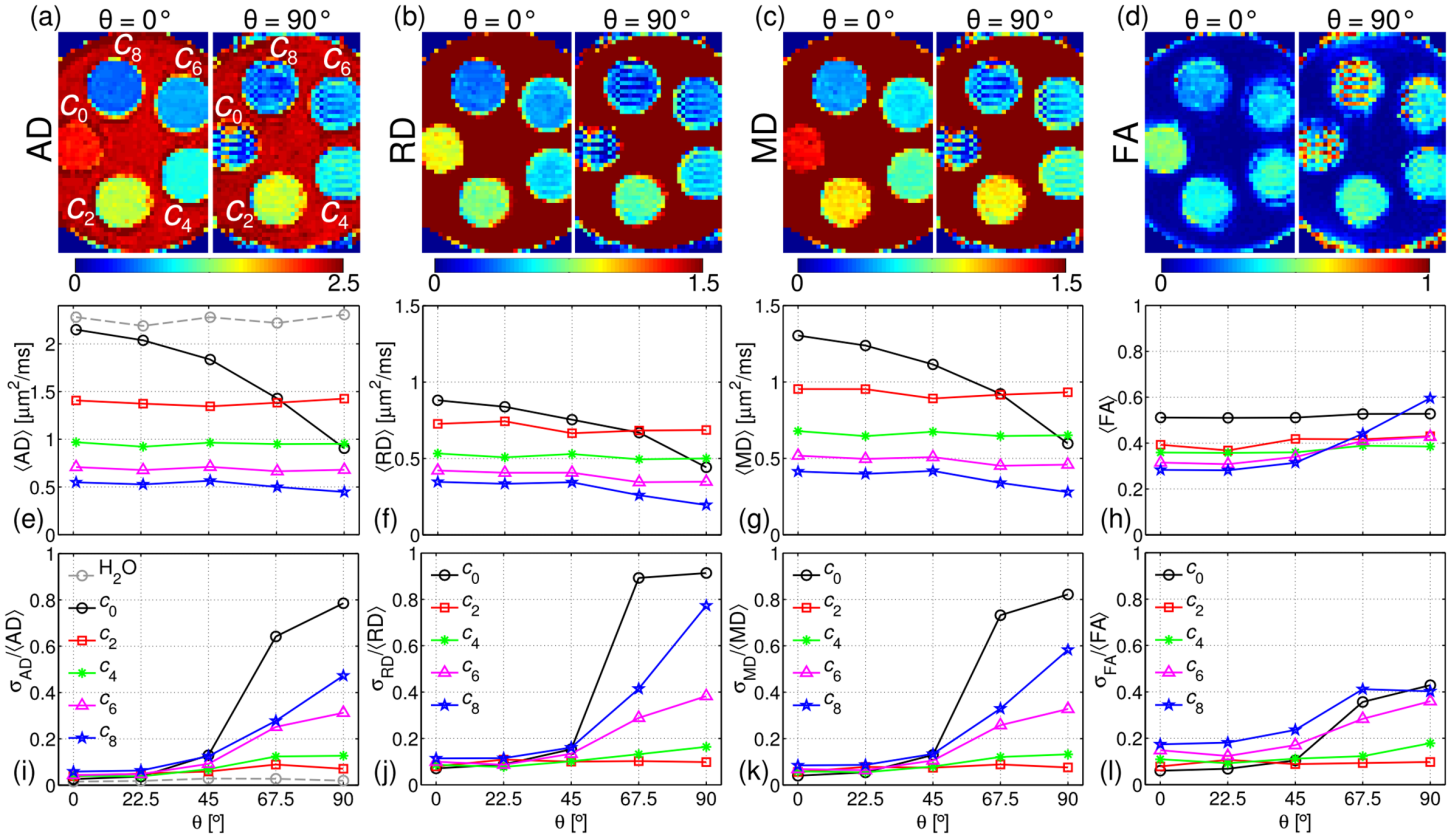
Maps of the DTI scalar parameters. AD (a), RD (b), MD (c) and FA (d) for the orientations *θ* = 0° (left-hand side) and *θ* = 90° (right-hand side). ROI-averaged values 〈*AD*〉 (e), 〈*RD*〉 (f), 〈*MD*〉 (g) and 〈*FA*〉 (h) and the normalised standard deviations σ_AD_/〈*AD*〉 (i), σ_RD_/〈*RD*〉 (j), σ_MD_/〈*MD*〉 (k) and σ_FA_/〈*FA*〉 (l). The bulk 〈*AD*〉 is shown as a reference by the dashed, grey line (e). The ROIs contain 1300±200 voxels.

Fig 4 shows the maps of the DTI metrics AD (a), RD (b), MD (c) and FA (d), for all five vials and for the two extreme angles *θ* = 0° (left-hand side) and 90° (right-hand side). One can see that all parameters and all vials show a rather homogeneous behaviour for *θ* = 0°. Whereas for *θ* = 90° severe image artefacts can be seen for all parameters and all vials except for the concentrations *c*_2_ and *c*_4_.

Fig 4e–4h demonstrate the angular dependence for the ROI-averaged values 〈*AD*〉, 〈*RD*〉, 〈*MD*〉 and 〈*FA*〉, respectively. The normalised standard deviations σ_AD_/〈*AD*〉, σ_RD_/〈*RD*〉, σ_MD_/〈*MD*〉 and σ_FA_/〈*FA*〉 are shown in Fig 4i–4l, respectively. It can be seen that for the concentration *c*_0_ all diffusion metrics are strongly underestimated when *θ* ≥ 45°. The rest of the concentrations show a qualitatively more stable behaviour for 0° ≤ *θ* ≤ 90°. However, Fig 4i–4l demonstrate that the normalised standard deviations for concentrations *c*_0_, *c*_6_ and *c*_8_, i.e., for susceptibility values far from the matching concentration, are strongly increased for *θ* ≥ 45°, whereas for *c*_2_ and *c*_4_ the change is virtually negligible in the whole angular range.

**Fig 5:**
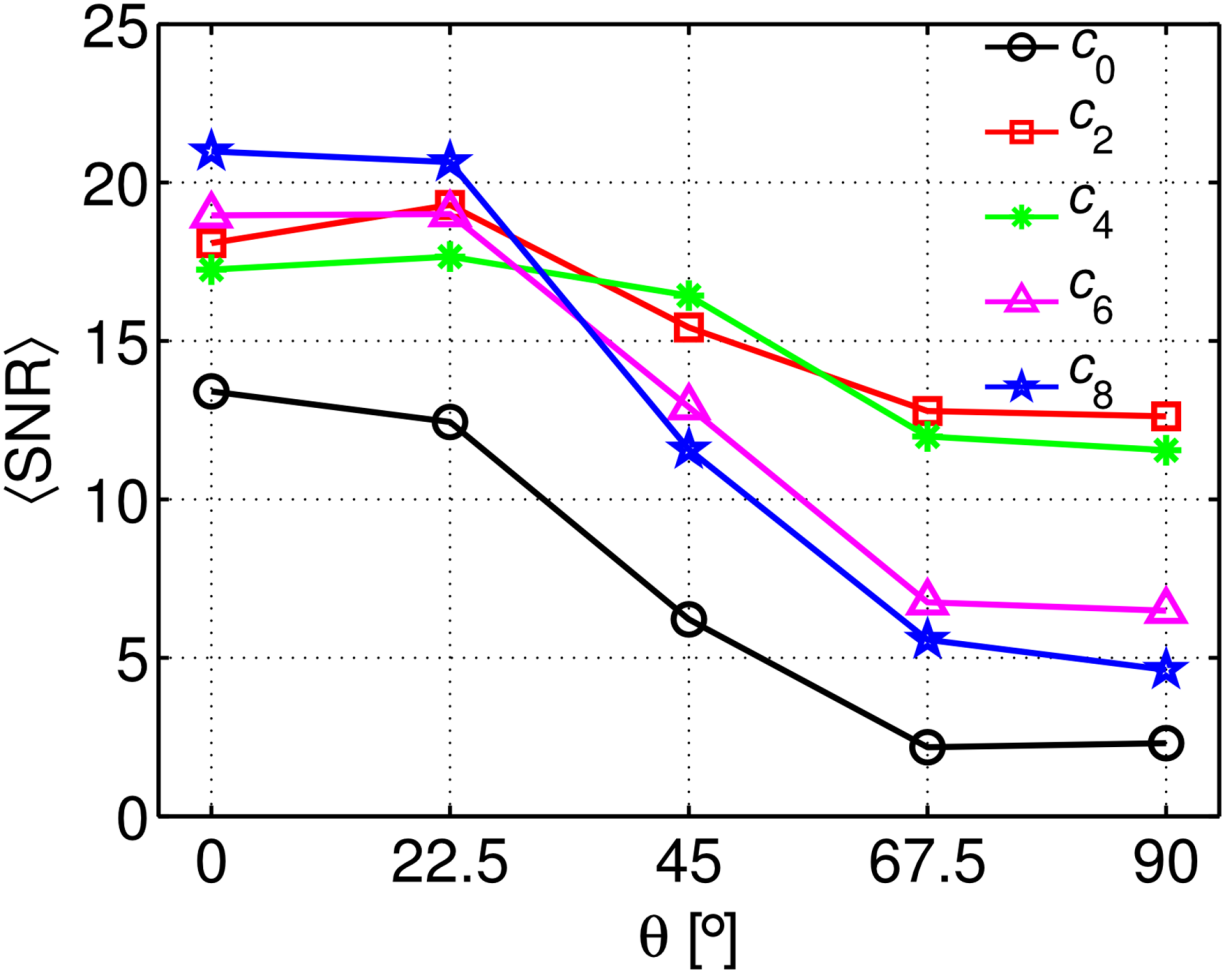
SNR values in the parallel-fibre phantoms. Calculated as SNR = *S*(b = 0)/σ, for 0° ≤ *θ* ≤ 90° in all concentrations.

The change of the SNR values, conventionally calculated as SNR = *S*(b = 0)/σ, for 0° ≤ *θ* ≤ 90° and all concentrations is shown in Fig 5. A strong decrease is seen for *c*_0_, *c*_6_ and *c*_8_ and *θ* ≥ 45° (~60% to ~80%), whereas for concentrations *c*_2_ and *c*_4_ the reduction is much smaller (~25%).

The results of the bulk measurements as well as of the parallel-fibre scenario clearly indicate *c*_3_ as the optimal concentration of the MgCl_2_·6H_2_O solution.

### Crossing-fibre phantom

**Fig 6:**
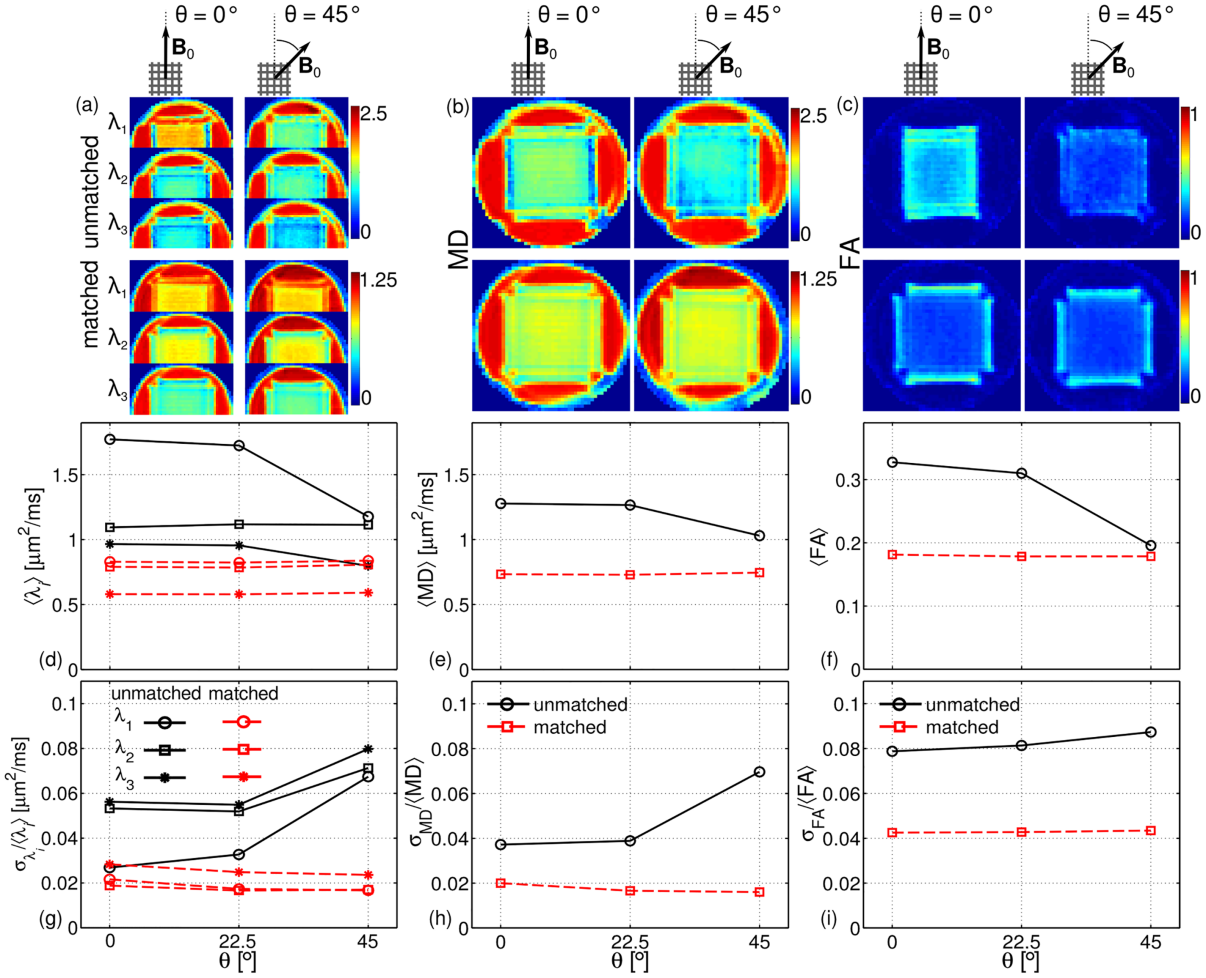
Maps of the DTI parameters in the crossing-fibre phantom. Eigenvalues *λ*_*i*_, *i* = 1,2,3 (a) and metrics MD (b) and FA (c) for the *unmatched* case (top) and the *matched* case (bottom). Two angular orientations are shown: *θ* = 0° on the left-hand side, and *θ* = 45° on the right-hand side. The angular dependence for the ROI-averaged values for 〈*λ*_*i*_〉 (d), 〈*MD*〉 (e) and 〈*FA*〉 (f). The corresponding normalised standard deviation values $\sigma_{\lambda_{\text{i}}}/\langle \lambda_{\text{i}}\rangle$ (g), σ_MD_/〈*MD*〉 (h) and σ_FA_/〈*FA*〉 (i), for the *unmatched* (black-solid lines) as well as for the *matched* (red-dashed lines) cases.

Fig 6 shows the maps of the diffusion tensor eigenvalues *λ*_*i*_, *i* = 1,2,3 (a) and the metrics MD (b) and FA (c) for the *unmatched* and the *matched* cases. Two angular orientations are shown: *θ* = 0° and *θ* = 45°. All maps show differences between the experimental setups with the phantom positioned at *θ* = 0° and *θ* = 45° for the *unmatched* case, whereas for the *matched* case the differences virtually disappear.

The angular dependence for the ROI-averaged values is shown in Fig 6d–6f for 〈*λ*_*i*_〉, 〈*MD*〉 and 〈*FA*〉, respectively. The corresponding normalised standard deviation values $\sigma_{\lambda_{\text{i}}}/\langle \lambda_{\text{i}}\rangle$, σ_MD_/〈*MD*〉 and σ_FA_/〈*FA*〉 are illustrated in Fig 6g–6i, respectively, for the *unmatched* as well as for the *matched* cases. For the *unmatched* case one finds *λ*_1_>*λ*_2_≈*λ*_3_ for *θ* = 0°, whereas *λ*_1_≈*λ*_2_>*λ*_3_ for *θ* = 45°, denoting a strong angular dependence of the shape of the diffusion tensor ellipsoid with regard to **B**_0_. On the contrary, for the *matched* case one observes that *λ*_1_≈*λ*_2_>*λ*_3_ for both orientations. Moreover, the normalised standard deviations for all parameters show higher values for the *unmatched* case than for the *matched* case.

**Fig 7:**
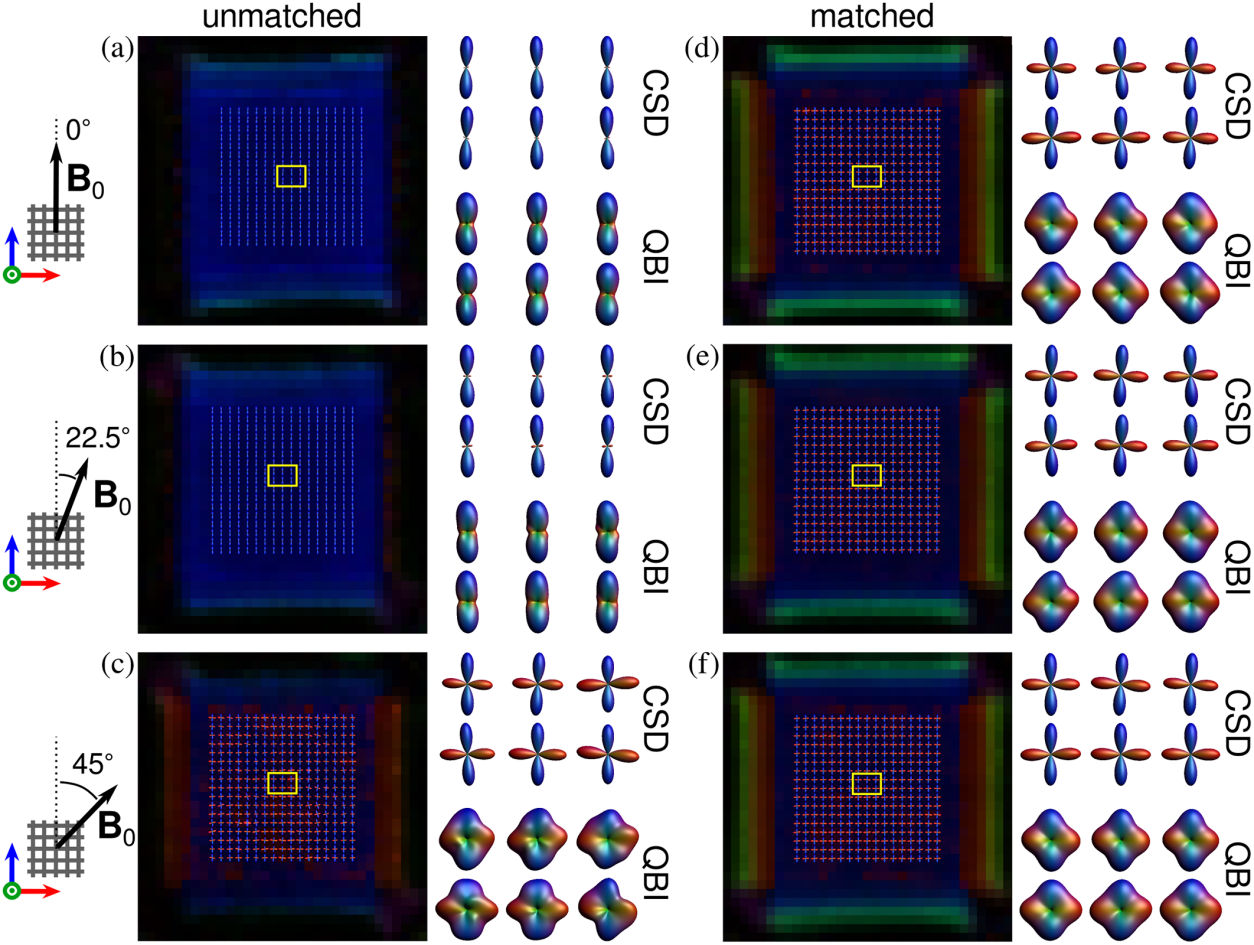
Performance of the CSD and QBI methods in the assessment of fibre orientations. The *unmatched* case is shown in the panel on the left-hand side for three phantom orientations (a-c). Similarly the *matched* case is shown in the panel on the right-hand side (d-f). The background image in each case corresponds to the colour-coded FA from conventional DTI.

The performance of the CSD and QBI methods is depicted in Fig 7. The *unmatched* case is shown in the panel on the left-hand side for three phantom orientations (Fig 7a–7c). Similarly the *matched* case is shown in the panel on the right-hand side (Fig 7d–7f). In the *unmatched* case and *θ* = 0° only the fibre population parallel to **B**_0_ is resolved, whereas the fibre population perpendicular to **B**_0_ is not visible (Fig 7a). Only for the orientation *θ* = 45° both fibre populations can be resolved (Fig 7c). Yet, for the *matched* case both fibre populations are clearly resolved, regardless of the phantom orientation with respect to **B**_0_ (Fig 7d–7f).

**Fig 8:**
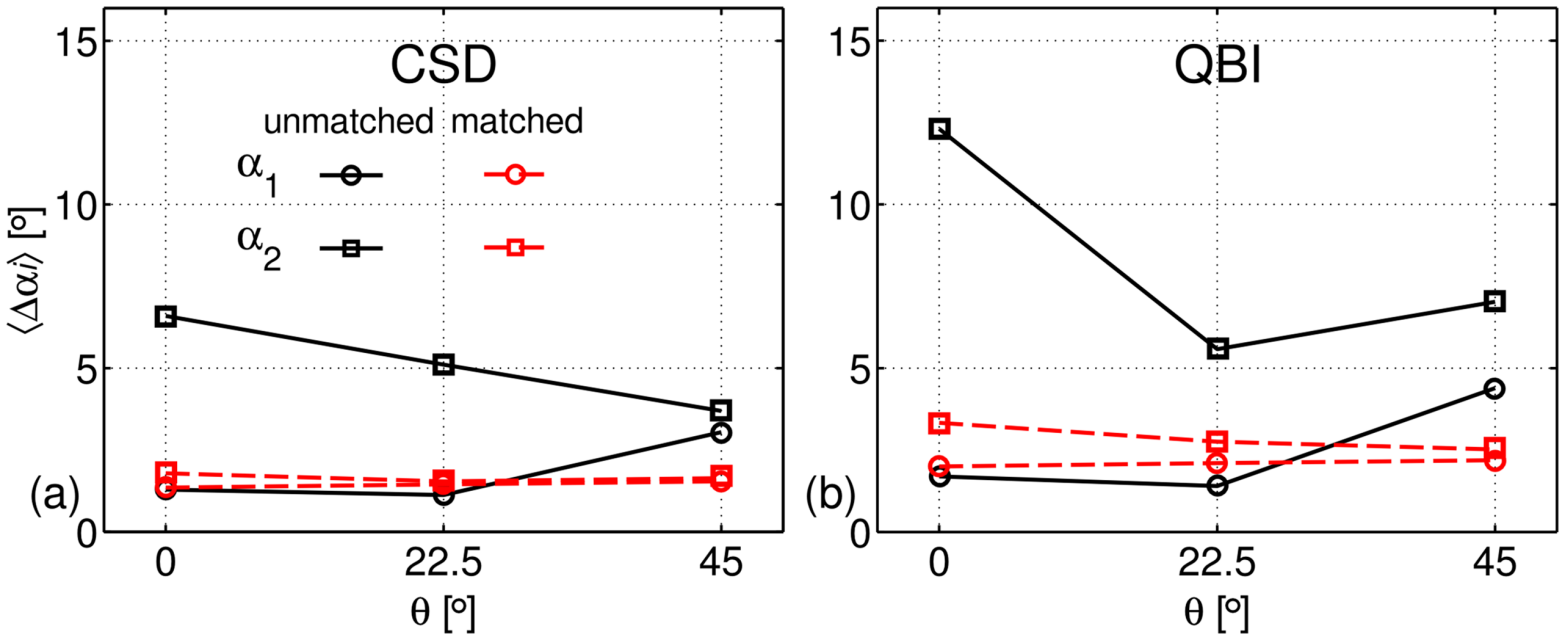
Dependence of the mean deviation angle Δ*α* with regard to *θ*. Δ*α* is evaluated for CSD (a) and for QBI (b) using Eq (6). Black lines correspond to the *unmatched case*, whereas red lines denote the *matched case*. Circles denote the main fibre direction and squares refer to the secondary fibre direction.

The dependence of the mean deviation angle, Δ*α*, with regard to *θ* as estimated using Eq (6), is depicted in Fig 8a for CSD and Fig 8b for QBI. For the *unmatched* case, one can clearly observe that Δ*α*_1_*>* Δ*α*_2_, for both CSD and QBI, whilst for the *matched* case, Δ*α*_1_ *≈* Δ*α*_2_. Both mean deviation values are lower than for the *unmatched* case.

## Discussion

### Bulk experiments

The characterisation of the bulk water NMR properties with increasing solute concentration reveals that the reduction in the bulk magnetic susceptibility entails other side effects, namely a decrease of the bulk longitudinal and transverse relaxation times, of the bulk diffusivity and of the proton density.

A decrease in *T*_1_ is beneficial as it allows for a reduction in repetition time without significantly affecting the SNR. On the other hand, a reduction in PD and *T*_2_ *a priori* gives rise to negative effects in DW MRI, since they contribute to diminish the SNR for a given TE. A decrease of ~10% in PD for *c*_3_ clearly implies a direct reduction in the SNR by the same amount. On the other hand, the observed decrease in *T*_2_ from 2600 ms for *c*_0_ to around 1040 ms for *c*_3_ introduces a minor reduction of the SNR values by ~6%, for an echo-time of 100 ms (typically used in DW MRI).

A decrease in the bulk diffusivity values from 2.2 μm^2^/ms for *c*_0_ to 1.13 μm^2^/ms for *c*_3_ was also measured. The bulk water diffusivity for increasing concentrations of MgCl_2_·6H_2_O has previously been investigated for the range of *c* = [0.24, 5.43] mol/l at 25°C using NMR [71]. The authors interpret such reduction in terms of the so-called two-phase model. Under this approach, water molecules are considered to be in fast exchange, on a nanosecond timescale, between two distinguishable water pools, namely the hydration layer at the ion Mg^2+^ and the bulk water. The hydration layer, composed by six H_2_O molecules, diffuses as a whole unit with a slower diffusivity, $D_{{\text{MgCl}}_{\text{2}}^{}}$, whereas the remaining pool, referring to the water beyond the hydration layer, is characterised by a faster diffusivity, *D*_bulk_. Thus, in the fast exchange limit, the mean diffusivity as measured with a PGSE sequence is given by $D_{{\text{H}}_{\text{2}}\text{O}}\left(c\right)=f\left(c\right)D_{{\text{MgCl}}_{\text{2}}}\left(c\right)+\left[1-f\left(c\right)\right]D_{\text{bulk}}\left(c\right)$, with *f*(*c*) being the mole fraction of the hydration layer [71]. In our case, the validity of the fast exchange limit is particularly important given that the DW signal attenuation will remain monoexponential, which is the desired behaviour for the bulk liquid used in diffusion phantoms. This behaviour was observed for all concentrations. Furthermore, the bulk diffusivity for *c*_3_ more closely resembles the free diffusion inside axonal bodies, i.e., ~1.4 μm^2^/ms [72].

### Parallel-fibre phantoms

According to Eq (3), the apparent RD can *a priori* be biased if the cross term is not taken into account in the signal model and the magnetic susceptibilities are not matched. However, given that the cross term can take negative and positive values, one needs to consider its behaviour in the whole volume of interest in order to predict the actual behaviour.

Our results show that there is an increasing underestimation of RD for increasing strength of the background gradients, i.e., increasing the angle of the fibre with respect to **B**_0_. Here, we refer to the works by Zhong et al. [38,39], in which a symmetric distribution for the cross term around zero in a heterogeneous, isotropic system was assumed. In their work, Zhong et al. postulate that the background gradients show a slow spatial change, i.e., each diffusing molecule experiences a constant background gradient during the time course of the experiment. Their results showed that the overall effect of the cross term is a decrease in the estimated diffusivity with increasing strength of the background gradients. In our parallel-fibre phantoms, the diffusion length *l*_d_, i.e., the root mean square displacements during *T*_E_ = 82 ms, was approximately 12 μm for *c*_0_ and 7.5 μm for *c*_8_ in the radial direction. On the other hand, considering the observed mean volume fibre fraction of ~0.64, one can estimate the structural length *l*_s_, i.e., the distances between the fibres in the interstitial space, to be in the order of 3 μm (assuming a simple hexagonal arrangement of the lattice as an example). Therefore given that *l*_s_ is shorter, the spins cross the pores (structural lengths) many times before dephasing and the magnetic field inhomogeneities are motionally averaged [73,74]. In other words, the water molecules experience a mean background gradient with slow spatial change and therefore, a similar argument to the one utilised by Zhong et al. can be used to explain the observed underestimation in RD in our work.

The apparent AD should instead ideally remain independent of the orientation as predicted by Eq (3). This can be observed for concentrations *c*_2_-*c*_8_. However, AD exhibits a strong underestimation for *c*_0_ at *θ* > 45°, as shown in Fig 4a and 4e. A possible explanation for that could be the fact that as a result of the strong reduction in *T*_2_ due to the strong background gradients, the SNR reaches very low values (Fig 5). As a consequence, the relative standard deviation σ_AD_/〈*AD*〉 reaches values of the order of 0.6 to 0.8 (Fig 4i), turning the estimation unreliable. However, given that all tensor elements were assessed using the asymptotically unbiased ML estimator, one can expect only a marginal influence of the low SNR in AD. On the other hand, a stronger influence is expected by the likely presence of some imperfections in the intra-voxel fibre alignment, which would result in an axial component of the background gradients. Moreover, the difference in magnetic susceptibility between the liquid inside the vial and the surrounding distilled water (see Fig 1, middle phantom), can create long-range field gradients that can *a priori* affect the estimation of AD.

Despite the aforementioned results, all parameters remained practically unchanged for the concentrations *c*_2_ and *c*_4_. Moreover, as a consequence of the minor reduction in the SNR values, the relative standard deviation for all parameters remains low across the whole angular range.

### Crossing-fibre phantoms

DTI analysis demonstrates that the ellipsoid representation of the diffusion tensor can appear strongly changed in the presence of background gradients. For the *unmatched case* and the orientation for *θ* = 0°, the tensor eigenvalues follow the relation *λ*_1_>*λ*_2_≈*λ*_3_ with *λ*_1_ being the diffusivity along the fibre population parallel to **B**_0_ (Fig 6a). This relation corresponds to the prolate shape of the ellipsoid, observed in systems with cylindrical geometry. In other words, only the fibre population parallel to **B**_0_ is observable. This result can be explained by the fact that the remaining fibre population suffers from a strong reduction in the SNR leading to a strong underestimation in the diffusivities, similar to that observed in the parallel-fibre phantom for *θ* = 90° (Fig 4e and 4f). Although the expected oblate shape of the ellipsoid for such fibre configuration, i.e., *λ*_1_≈*λ*_2_>*λ*_3_, is recovered for the orientation *θ* = 45°, the absolute values of *λ*_*i*_ are still biased by the background gradients. The effect on the estimation of the fibre populations can be more clearly observed in the CSD and QBI analysis in Fig 7a–7c, where both fibre populations are recovered only for *θ* = 45°.

On the other hand, the dependence of the tensor eigenvalues on the orientation with respect to **B**_0_ is completely suppressed in the *matched* case, as shown in Fig 6. Similarly, both fibre populations are clearly identified using CSD and QBI, independently of the phantom orientation. This is even more emphasised by Fig 8, showing that the angular deviations for both populations are similar and practically independent of the phantom orientation.

Notwithstanding the advantages of the improved design of the phantom, one recognises several limitations of the fibres themselves regarding their capability for mimicking white matter tissue properties. One of their intrinsic limitations is that, being rod-like fibres, they resemble the diffusion properties of only the extracellular space in the white matter tissue. Moreover, white matter fibres are not perfect cylinders, but their profile is modulated by the Ranvier nodes, a fact that is not reflected in Dyneema fibres.

## Conclusions

In this work we have assessed one of the crucial points in the construction of anisotropic diffusion fibre phantom, namely the necessary independence of the diffusion metrics on the orientation of the phantom in the static magnetic field. We showed that susceptibility differences between fibre and liquid are responsible for the angular dependence of the diffusion response signal and consequently of the diffusion parameters and the reconstruction of fibre tracts.

It is demonstrated that an experimentally determined concentration of 1.87 mol/l MgCl_2_·6H_2_O dissolved in distilled water removes the susceptibility difference between the liquid and the fibres, and hence eliminates the microscopic gradient fields in the interstitial space. Susceptibility matching leads to complete angular independence of the diffusion metrics in complex anisotropic diffusion phantoms. This, in turn, allows one to conduct DW MRI experiments without the need to consider the geometric alignment of the involved structures with respect to the direction of static magnetic field. Moreover, our findings are of further interest for the investigation of diffusion in complex scenarios involving susceptibility differences and local microscopic field gradients.

## Supporting information

S1 File Archive containing results. Datasets are indexed and described in CONTENTS.txt.
(ZIP)
